# Flavor Evolution and Quality Changes in Hot-Pressed Peanut Oil: Impact of Roasting Temperature and Storage Time

**DOI:** 10.3390/foods14223945

**Published:** 2025-11-18

**Authors:** Guang Yang, Zhiran Zhang, Mengkai Liu, Ziyan Zhang, Gaoyuan Kong, Sen Zhou, Shengxin Li, Jie Sun

**Affiliations:** 1 College of Life Sciences, Qingdao University, Qingdao 266071, China; sunlight_yangg@163.com (G.Y.); zzrzgj@163.com (Z.Z.); liumengkaikai@163.com (M.L.); zhangziyan77@126.com (Z.Z.); jason_zhousen@163.com (S.Z.); lsxy0215@163.com (S.L.); 2 Qingdao Agricultural Technology Storage Extension Station, Qingdao 266071, China; qdnjzx@qd.shandong.cn

**Keywords:** peanut oils, flavor, GC-MS, GC-IMS, storage period

## Abstract

Storage time significantly influences the aroma quality of peanut oil. In this study, gas chromatography–mass spectrometry (GC-MS) and gas chromatography–ion migration spectrometry (GC-IMS) were used to analyze the volatile flavor compounds of hot-pressed peanut oil baked at two temperatures (140 °C and 160 °C, denoted as OPO and RPO, respectively) during storage. The two methods detected 80 and 76 volatile flavor compounds, respectively, and principal component analysis (PCA) revealed clear distinctions between OPO and RPO during the storage period. Ten key aroma compounds were identified based on relative odor activity value (ROAV) analysis, including 3-methylbutanal, hexanal, heptanal, octanal, benzeneacetaldehyde, 3-ethyl-2,5-dimethylpyrazine,2-ethyl-6-methylpyrazine, acetylpyrazine, 2-methoxy- 4-vinylphenol, and acetic acid. During storage, the degradation and transformation of flavor compounds were more pronounced in RPO than in OPO. Physicochemical analyses showed increased acid and peroxide values, concomitant with decreased vitamin E and phytosterol content. Notably, these parameters changed more slowly in OPO than in RPO, confirming that OPO maintained superior quality over time. Correlation analysis suggested that changes in the aldehyde and pyrazine contents are key indicators of flavor and quality evolution in peanut oil during storage. This study provides insights into how baking temperature and storage time affect peanut oil aroma, elucidating the mechanisms of flavor variation and offering a theoretical basis for optimizing the flavor and quality of hot-pressed peanut oil.

## 1. Introduction

*Peanut* (*Arachis hypogaea* L.) is one of the world’s most important oil crops, with an oil content of 44–56% [1]. Global production in 2022–2023 reached 49.36 million metric tons (MMT), with peanut oil production at 6.23 MMT [2]. Peanut oil is an edible oil of high nutritional value, containing plant sterols, vitamin E, resveratrol and other natural antioxidants that can neutralize free radicals and reduce cellular oxidative damage [3]. It is popular among consumers for its distinctive flavor, and its consumption has increased in recent years [4]. 

Flavor is a critical component of the sensory quality of plant oils and largely determines consumer preference and purchasing intention. Peanuts must be heat-treated or roasted before oil extraction to inactivate enzymes, denature proteins, impart flavor and aroma to the oil, and promote the release of oil components [5]. Hot-pressed peanut oil develops more intense nutty, burnt, rancid, oily, and grassy notes through complex chemical reactions, such as the Maillard reaction, Strecker degradation, and lipid oxidation [4,6]. These flavor notes are derived from compounds such as aldehydes, alcohols, furans, and pyrazines [7]. Two main types of hot-pressed peanut oil are consumed in China, OPO and RPO, both of which have large consumer bases. RPO exhibits a heavy burnt-paste flavor, whereas OPO has a pronounced nutty aroma; these flavor differences result from variations in processing conditions (specifically the baking temperature). To our knowledge, no study has characterized the compositional differences in flavor between these two types of peanut oils. 

Numerous studies have demonstrated that baking temperature significantly influences the composition and concentration of flavor compounds in peanut oil. Zhang et al. observed that peanut oil pressed at 100–150 °C developed an increasingly pronounced nutty flavor as the roasting temperature increased [8]. Understanding how roasting conditions affect flavor can inform the optimization of the production process, intensifying desirable flavor characteristics and enhancing consumer appeal. However, storage time also affects the flavor and physicochemical properties of plant oils. As flavor compounds are highly volatile, their composition and concentration change during storage, thereby altering the overall flavor profile of peanut oil. To date, no study has systematically investigated the temporal evolution of flavor compounds in peanut oil during its storage. Such research is important for optimizing production processes and preserving the peanut oil flavor during storage.

Descriptive sensory analysis is an effective tool for relating product attributes to sensory perception [9]; however, its results are inherently subjective and cannot reveal much about the molecular basis of flavor. Analytical techniques such as solid-phase microextraction coupled with gas chromatography–mass spectrometry (GC-MS) and gas chromatography–ion migration spectrometry (GC-IMS) enable the detailed separation and identification of volatile compounds. GC-IMS is a novel, rapid, and visual technique for flavor profiling, although it has limitations in quantitative analysis [10]. GC-MS, on the other hand, provides both qualitative and quantitative analyses but is less straightforward for visual comparison across sample groups [10]. By combining these methods, more comprehensive flavor analysis can be achieved, leveraging their complementary strengths in terms of convenience, efficiency, and sensitivity [7,11]. This integrated approach can more fully characterize the volatile compounds in peanut oil over the storage time and elucidate the relationship between these compounds and sensory attributes [12].

As storage time increases, peanut oil undergoes oxidation, resulting in rancidity and a pungent off-flavor [13]. Vitamin E and phytosterols, which are abundant in peanut oil, are important micronutrients and antioxidants; their synergistic effects can inhibit the oxidation in foods, thereby extending shelf life and improving overall oil quality [14]. Studies have shown that as the peanut roasting temperature increases from 120 °C to 160 °C, while the acid value (AV), peroxide value (PV), and phytosterol content of peanut oil increase, whereas the vitamin E content remains relatively unchanged [15]. High-temperature roasting imparts a rich, nutty flavor.

However, the temporal evolution of flavor compounds and their correlation with key physicochemical properties (e.g., AV, PV, vitamin E, and phytosterols) during the storage of these oils remain unclear. Therefore, this study aimed to employ GC-MS, GC-IMS, and sensory evaluation to characterize the flavor profiles of OPO and RPO and monitor their changes during 12 months of storage. The relationships between typical flavor compounds, relative odor activity values (ROAVs), and quality parameters were examined to elucidate the mechanisms of flavor variation and provide a theoretical basis for optimizing flavor and quality.

## 2. Materials and Methods

### 2.1. Materials

A DVB/CAR/PDMS solid-phase microextraction (SPME) fiber (50/30 μm, 2 cm) was purchased from Sigma-Aldrich (Supelco, Bellefonte, PA, USA). Headspace vials (20 mL) fitted with 18 mm magnetic PTFE/silicone caps were purchased from Agilent Technologies Inc. (Palo Alto, CA, USA). C7–C30 n-alkanes and C4–C9 n-ketone standards were obtained from Sigma-Aldrich Chemical Co. (St. Louis, MO, USA), 4-Nonanol (99%, AR) were purchased from Aladdin (Shanghai, China).

### 2.2. Seed Roasting and Oil Extraction

One kilogram of peanut seeds was divided into two batches and roasted at 140 °C and 160 °C for 30 min using a continuous circular monolayer roasting machine (Jiangsu Maisi Machinery Equipment Co., Ltd., Suzhou, China). The obtained oils were immediately bottled in 100 mL clear glass bottles and stored at ambient temperature (approximately 25 °C) under normal laboratory lighting conditions for the duration of the study. The roasted seeds were then pressed at room temperature using a Westinghouse (Pittsburgh, PA, USA) to obtain peanut oils, which were labeled OPO (from the 140 °C roast) and RPO (from the 160 °C roast). The obtained oils were immediately bottled and stored at ambient temperature. The oils were bottled in 250 mL clear glass bottles and stored at ambient temperature (approximately 25 °C) under normal laboratory lighting conditions, simulating a typical shelf storage environment. Samples were collected at 0, 6, and 12 months of storage and designated OPO-0, RPO-0, OPO-6, RPO-6, OPO-12, and RPO-12 for subsequent analyses.

### 2.3. Sensory Evaluation

Quantitative descriptive analysis (QDA) was used to evaluate the flavor attributes of the peanut oil samples. A panel of 20 trained evaluators (10 males and 10 females, aged 20–25 years) from the Sensory Evaluation Laboratory of Qingdao University participated in this study. The assessors underwent a standardized training period of two weeks. During this time, they were familiarized with the target flavor attributes using reference standards (e.g., hexanal for grassy, 2-acetylpyrazine for nutty) and practiced intensity scoring. All panelists were healthy and did not have any olfactory or gustatory impairments. Flavor intensity was scored on a 0–10 scale (0 = no sensation, 1 = very weak, 5 = moderate, and 10 = very strong), and the scores were recorded for analysis (Table 1) [16].

The study adhered strictly to the National Institutes of Health Guide for the care and use of laboratory animals and was approved by the Ethics Committee Medical College of Qingdao University(Ethics Review Number: QDU-HEC-2025466). During the experiment, the panelists refrained from smoking, eating, or using strongly scented products for at least 1 h prior to and during the testing. All panelists were trained in the sensory evaluation protocol and briefed on the evaluation procedures before testing.

### 2.4. Headspace Solid-Phase Microextraction

Volatile compounds in peanut oils were extracted using headspace solid-phase microextraction (HS-SPME) before chromatographic analysis

#### 2.4.1. HS-SPME/GC-MS Analysis

Modified appropriately from the previous study [17], 3 mL of each peanut oil sample was added to a headspace bottle by solid phase microextraction to extract volatile compounds, and DB-WAX UI capillary column (30 m × 0.250 mm × 0.25 μm) was used. After adsorption, the fiber was retracted and inserted into the injector port of a gas chromatography–mass spectrometry (GC-MS) system (Thermo Fisher Scientific, Waltham, MA, USA) for thermal desorption (~10 min). Volatile compounds were separated on a DB-WAX UI capillary column (30 m × 0.25 mm i.d., 0.25 μm film thickness; Agilent Technologies) using helium (>99.999% purity) as the carrier gas at a flow rate of 1 mL/min. The GC oven temperature was programmed from 50 °C (held for 5 min) to 120 °C at 3 °C/min, then ramped to 250 °C at 5 °C/min, and held for 5 min. The mass spectrometer was operated in electron impact mode (70 eV) with a scan range of m/z 30–550. The transfer line and ion-source temperatures were set at 280 °C and 250 °C, respectively. The compounds were tentatively identified by matching their mass spectra with those in the NIST17 library (similarity > 85%). Major volatiles were quantified using an internal standard method (5 μL of 0.8 mg/mL 4-nonanol).

#### 2.4.2. Relative Odor Activity Value (ROAV)

The relative odor activity values (ROAVs) for each volatile compound were calculated by dividing the measured concentration by the corresponding odor threshold value. Compounds with ROAV ≥ 1 were considered odor-active and significant contributors to the overall aroma of peanut oil [17].

ROAV = C/T

where C is the concentration of the volatile compound (mg/kg), T is its odor threshold in oil (mg/kg).

#### 2.4.3. GC-IMS

For GC-IMS analysis, 1.5 mL of each peanut oil sample was placed in a 20 mL headspace vial and equilibrated at 80 °C for 15 min with agitation (300 rpm). A 200 µL sample of the headspace was automatically injected into the GC-IMS system using an injector temperature of 85 °C. Separation was achieved on a DB-WAX metal capillary column (15 m × 0.53 mm, 1.0 µm film thickness) maintained at 60 °C. Ultra-high-purity nitrogen (99.999% purity) was used as the carrier gas with the following flow program: 2 mL/min for 2 min, ramping to 10 mL/min over 8 min, then to 100 mL/min over 10 min, and maintained at 100 mL/min for 20 min. The drift gas (nitrogen) was set at 150 mL/min, and the IMS detector was operated at 45 °C. Each GC-IMS analysis was performed in triplicate. The retention indices (RIs) of the volatile compounds were calculated using the C4–C9 n-ketone series as an external reference. Volatiles were identified by comparing their RI and drift times with those of reference standards in the user-built database, as well as the NIST 17 library and GC-IMS database (G.A.S GmbH, Dortmund, Germany). Data processing was performed using the LAVsoftware 0.78(G.A.S., Dortmund, Germany) [18]. 

### 2.5. Physical and Chemical Property Analysis

To assess the storage stability of peanut oil, various physical and chemical properties were measured after 0, 6, and 12 months of storage. For example, the color parameters of the oils were determined using a HunterLab colorimeter (USA Hunter Associates Laboratory, Inc., Reston, VA, USA). Specifically, the color coordinates L* (bright to dark), a* (red to green), and b* (yellow to blue) of oxidized (OPO) and RPO were recorded [19]. At the same time points, additional physicochemical properties, including peroxide value, acid value, phytosterol content, and vitamin E content, were determined as described below.

#### 2.5.1. Peroxide Value

The peroxide value (PV) was determined by iodometric titration according to the Chinese National Standard GB/T 5009.227—2016 [20]. Briefly, 0.5 g of oil was mixed with 5 mL of a glacial acetic acid-chloroform solution (3:2 *v*/*v*). Then, 50 μL of a saturated potassium iodide solution was added to the mixture. The reaction was allowed to proceed at room temperature for 5 min, and then 5 mL of distilled water and 25 μL of a starch indicator solution (1 g per 100 mL) were added. The resulting mixture was titrated with 0.10 M sodium thiosulfate until a persistent endpoint was reached (the blue color of the starch indicator disappeared). Blank titrations were performed in parallel, without oil samples [21,22]. The peroxide value was calculated using the following equation:

PV = [(V − V_0_)C × 1000]/m

where m is the mass of oil (g), C is the concentration (mol/L) of the sodium thiosulfate solution (M), V is the volume (mL) of sodium thiosulfate used for the sample, and V_0_ is the volume (mL) of sodium thiosulfate used for the blank.

#### 2.5.2. Acid Value

The acid value (AV) was determined using the cold solvent-indicator titration method described in the Chinese National Standard GB/T 5009.229—2016 [20]. In this procedure, 20 g of peanut oil was weighed and dissolved in 50 mL of an ether–isopropanol mixture (1:1, *v*/*v*) containing a few drops of phenolphthalein. The solution was shaken gently and titrated with 0.10 M sodium hydroxide solution using a burette. The titration endpoint was reached when the solution turned pale pink and retained its color for a minimum of 15 s. The volume of KOH solution consumed in the sample titration was recorded as V, and the volume consumed in a blank titration (without oil) was recorded as V_0_. The acid value (mg KOH per g oil) was calculated using the following formula:

XAV = (V − V_0_) × c × 56.1 m × 100%

where V and V_0_ are in milliliters, c is the molar concentration (mol/L) of the KOH solution, 56.1 is the molar mass of potassium hydroxide (g/mol), and m is the mass of the extracted oil (g).

#### 2.5.3. Analysis of Phytosterol Content

Phytosterol content was determined using gas chromatography–mass spectrometry (GC-MS), with a slight modification of the method of Liang et al. [23]. The oil samples were saponified using an ethanol-potassium hydroxide solution to obtain an unsaponifiable fraction. An Agilent 7890B gas chromatograph (Agilent, Santa Clara, CA, USA) equipped with a DB-5MS capillary column (15 m × 0.25 mm × 0.25 μm; Agilent) was used for analysis. The injector temperature was maintained at 320 °C. The column was initially held at 240 °C and then heated at 4 °C/min to 255 °C. Hydrogen was used as the carrier gas at a linear velocity of 36 cm/s. The injection volume was 1 μL, and a split ratio of 1:20 was employed. Phytosterols were identified by comparing their retention times with those of standard compounds (e.g., betulin, Supelco, Bellefonte, PA, USA). Quantification was performed using calibration curves constructed from standard curves. All analyses were performed in triplicate, and the results are reported as mean ± standard deviation (SD).

#### 2.5.4. Vitamin E Content Detection

Vitamin E (tocopherol and tocotrienol isomers) content was determined by high-performance liquid chromatography (HPLC) following a modified procedure of Martakos et al. [24]. Each sample (100 g oil) was dissolved in 9 mL of 2-propyl alcohol and filtered through a 0.22 μm membrane filter. A 20 μL aliquot of the filtrate was injected into an Agilent 1200 series HPLC system (Agilent, Santa Clara, CA, USA) equipped with an autosampler (G1329A), column thermostat (G1330B), binary pump (G1312A), and a diode-array detector (G1315D). Separation was achieved on a Waters Spherisorb ODS column, (2250 mm × 4.6 mm, diameter 5 μm) purchased from Waters (Milford, MA, USA). The mobile phase consisted of methanol (solvent A) and acetonitrile (solvent B) with the following gradient: 0–7 min, 50% A (isocratic); 7–12 min, linear increase to 100% A; 12–15 min, hold at 100% A; and 15–27 min, linear decrease to 50% A, and stabilization for 3 min. Then, lower (A) to 50% for another 12 min. The flow rate was 1.0 mL/min, and detection was performed at 295 nm. Vitamin E compounds were identified by retention time and UV spectra and quantified using external standard calibration curves. 

### 2.6. Statistical Analysis

All experiments were conducted more than three times unless otherwise stated. Data are expressed as mean ± standard deviation. One-way analysis of variance (ANOVA) followed by Duncan’s multiple range test was performed using SPSS version 27.0 (SPSS Inc., Chicago, IL, USA). Differences were considered statistically significant at *p* < 0.05. Principal component analysis (PCA) was performed using SIMCA software (version 14.1; Umetrics, Umeå, Sweden). Correlation analysis of flavor compounds was performed using Origin software (version 2024b).

## 3. Results and Discussion

### 3.1. GC-MS Analysis

#### 3.1.1. Identification of Volatile Components

GC-MS analysis identified 80 volatile compounds, which were categorized into 17 pyrazines, 16 aldehydes, 8 ketones, 7 acids, 6 alcohols, 5 amines, 5 phenols, 4 esters, 4 alkanes, 3 pyridines, 2 furans, 1 pyrrole, 1 nitrile, and 1 sulfide (Table S1).The main contributors to the flavor of the two oils were pyrazine, aldehydes (OPO), acids (RPO), pyrrole (RPO), ketones, pyridine, and furan (RPO). Other volatiles were present at much lower concentrations and had a minor impact on overall flavor. Notably, OPO contained two additional types of volatile compounds(2,5-dimethyl-pyrazine, 2,6-dimethyl-pyrazine) compared to RPO, although the concentrations of many of these compounds were lower than those in RPO. This difference was likely due to the higher roasting temperature used for RPO, which resulted in a more intense initial flavor. Baking at 160 °C accelerated the Maillard reaction, causing charring of the peanut surface, promoting amino acid decarboxylation, and increasing the oxidation rate [25]. Heterocyclic compounds (including pyrazines, pyridines, and furans) have been reported to produce nutty flavors, whereas aldehydes primarily confer fatty and grassy notes. Acids impart cheesy and sweaty flavors; esters contribute fruity and coconut-like aromas; phenols impart clove-like, smoky, and woody flavors; and sulfur-containing compounds generate a sulfurous flavor in the oil [26]. The combined effects of these flavor compounds resulted in the distinct flavor profiles of OPO and RPO.

**Figure 1:**
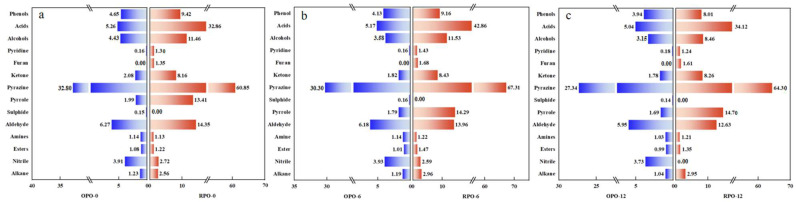
Changes in the concentrations of different types of volatile compounds in OPO and RPO during storage.

Based on previous research, the possible formation mechanisms and synthetic pathways of these volatiles were also analyzed. During heating, the unsaturated fatty acids in peanut oil undergo Strecker degradation and thermal oxidation, generating aldehydes and ketones [27]. The concentrations of aldehydes were 6.27 mg/kg in OPO and 14.40 mg/kg in RPO, while those of ketones were 2.08 mg/kg in OPO and 6.81 mg/kg in RPO. Among these, furfural (1.79 ± 0.09 mg/kg in OPO and 5.77 ± 0.29 mg/kg in RPO) and hexanal (1.26 ± 0.11 mg/kg in OPO) were the most abundant. 2-Acetone-1-acetoxy exhibited the highest concentration in OPO (0.37 ± 0.04 mg/kg), whereas hydroxyacetone was most concentrated in RPO (2.30 ± 0.11 mg/kg). As the baking temperature increased, the Maillard reaction intensified, leading to the condensation of aldehydes and ketones into heterocyclic compounds (pyrazines, pyridines, pyrroles, and furans) and phenolic compounds [28]. Proportionally, pyrazines represented the largest fraction of volatiles, accounting for 60.85% of the total volatile compounds in RPO and 32.80% in (OPO) (Figure 1). Pyridine and pyrrole are typical products of the Maillard reaction in amino acid-sugar systems, with 2-acetylpyrrole being particularly prevalent [29]. In this study, 2-acetylpyrrole was detected in both OPO and RPO, and its concentration was 60% higher in RPO than in OPO. Furan compounds are primarily formed via lipid peroxidation and carbohydrate degradation. In this study, 2-acetylfuran (0.97 ± 0.07 mg/kg) was identified as a representative furan and was found only in the RPO. Phenolic compounds tend to darken the color of oil and impart a sour taste; 2-methoxy-4-vinylphenol is the most prominent phenolic compound observed [30]. Notably, sulfur-containing compounds were only detected in OPO at a concentration of 0.15%, and showed no significant changes during storage. These compounds were the main source of OPO’s grassy aroma. This observation may be attributed to the degradation of sulfur compounds in RPO during baking at 160 °C [15].

Figure 1 illustrates the changes in the types and concentrations of flavor compounds during the storage period. Significant differences were observed between the two peanut oils in terms of the concentrations of the identified volatiles (*p* < 0.05). Alcohols, ketones, phenols, and alkanes exhibited a decreasing trend during storage in both oils, whereas nitriles increased from month 1 to month 6 of storage and then decreased from month 6 to month 12. This behavior can be attributed to the fact that heating significantly promotes the thermal decomposition of certain compounds (such as glucosinolates), leading to a transient increase in their concentration [26]. In OPO, the contents of pyrazine, pyrrole, aldehydes, esters, and acids decreased over time, whereas the levels of pyridine and sulfides remained relatively unchanged. In contrast, in RPO, pyrazines, aldehydes, esters, acids, and furans increased from month 1 to month 6 and then decreased from month 6 to 12. These patterns were attributed to the ongoing Strecker degradation reaction during storage, in which amino acids react with α-dicarbonyl compounds (losing a CO_2_ molecule) to form new flavor compounds. This reflects the higher baking temperature of RPO, which intensified the Maillard reaction during production, resulting in more pronounced Strecker degradation during storage than that in OPO.

GC-MS detected numerous prominent volatile compounds in peanut oils produced under different baking conditions. The observed differences in the concentrations of these volatiles in OPO and RPO correspond to the differences in sensory evaluation and flavor between the two oils. These findings underscore the need for further characterization and quantification of these volatile compounds to fully evaluate their impact on the overall flavor profile.

#### 3.1.2. ROAV Analysis

The important aromatic compounds identified by GC-MS were quantified, and their respective ROAVs (relative odor activity values) were calculated. ROAVs were defined as the ratio of a compound’s concentration to its odor threshold, reflecting the contribution of flavor components to the overall aroma (Table 2) [27]. This concept, which combines flavor threshold data with concentration information, is crucial for evaluating the impact of individual compounds on the overall flavor [28]. In general, compounds with 0.1 ≤ ROAV ≤ 1 are regarded as odor-active, whereas compounds with ROAV ≥ 1 are considered key flavor-active substances [29]. This classification indicates that compounds with ROAV ≥ 1 occur at concentrations above their odor thresholds and can express their characteristic aromas [30]. Based on the ROAV analysis, ten key flavor compounds were identified in hot-pressed peanut oil during storage: 3-methyl-butanal, hexanal, heptanal, octanal, benzeneacetaldehyde, 3-ethyl-2,5-dimethylpyrazine, 2-ethyl-6-methyl-Pyrazine, acetylpyrazine, 2-Methoxy-4-vinylphenol, and acetic acid.

The initial total ROAV of the volatile components in RPO was greater than that in OPO, indicating an enhanced initial flavor intensity (Table 2). The total ROAV of the volatile compounds in both oils gradually decreased with increasing storage time. After six months of storage, the characteristic flavor compounds in OPO remained relatively stable, with minimal changes in composition, whereas RPO showed accelerated flavor degradation and significant changes in compound levels (*p* < 0.05). This difference can be attributed to the high-temperature processing of RPO (160 °C), which promotes Maillard reaction-driven matrix changes, accelerating flavor loss.

**Figure 2:**
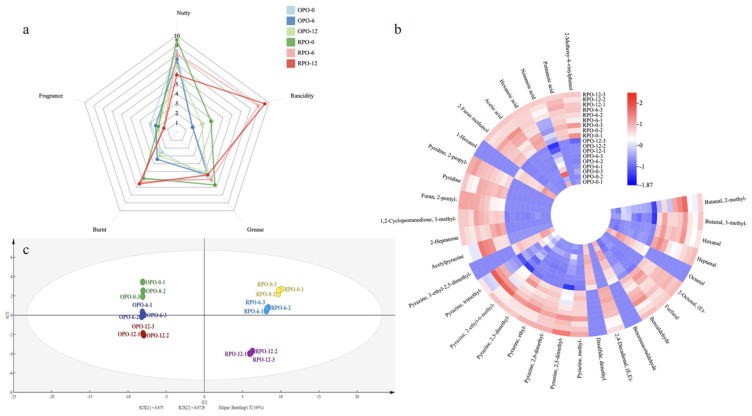
Flavor evaluation of OPO and RPO during storage period (**a**) sensory evaluation. (**b**) thermal map analysis of substances with ROAV > 1. (**c**) PCA.

A hierarchical clustering heat map of the ROAVs was generated. In this map, brighter colors indicate higher relative intensities, highlighting significant differences (*p* < 0.05) in the distribution and relative abundance of volatile components in peanut oils extracted at different roasting temperatures (Figure 2b). The results showed that aldehydes are among the most sensitive indicators of lipid oxidation [29,31]. This is due to their high content in peanuts (6.27–14.40%), diversity (11 types), and low ROAV threshold, which significantly impacts the overall flavor [7]. After one month of storage, seven compounds with ROAV ≥ 1 were detected in OPO. The core odorants included 3-methyl-butanal (ROAV = 2464.08 ± 121.39) and hexanal (ROAV = 899.64 ± 63.31), which mainly contributed to fatty and grassy notes [29,32]. Eight compounds with ROAV ≥ 1 were detected in the RPO. In addition to these two, heptanal (ROAV = 706.94 ± 30.38) was identified as a core odorant imparting a pungent note and is a known product of oil oxidation. Pyrazines have relatively low odor thresholds, and those with ROAV ≥ 1 contribute most strongly to the flavor of baked peanut oil. 6 Six Pyrazine compounds with ROAV ≥ 1 were identified in OPO, with 3-ethyl-2,5- dimethylpyrazine as the core aroma (ROAV = 668.41 ± 49.79), and eight pyrazine compounds with ROAV ≥ 1 were identified in RPO, with acetylpyrazine as the core aroma (ROAV = 2071.32 ± 25.08). These compounds are the main sources of roasted and nutty notes in peanut oil [33]. Acidic compounds can impart off-odors such as rancidity. Among the volatile acids, acetic acid had the greatest influence on flavor in both OPO and RPO (ROAV = 338.38 ± 19.95 vs. 2042.87 ± 93.93). However, with increasing storage time, the total acid concentration in RPO became significantly higher than that in OPO. This can be attributed to the formation of free amino acid by-products via Strecker degradation when baking exceeds the optimal temperature, which is a main factor in sensory deterioration [34]. Additionally, the ROAV of 2-methoxy-4-vinylphenol was significantly higher in RPO (447.28 ± 54.54) than in OPO (201.02 ± 17.55), contributing primarily to smoky and spicy notes.

### 3.2. GC-IMS Analysis

#### 3.2.1. Volatile Compounds Identification in OPO and RPO

**Figure 3:**
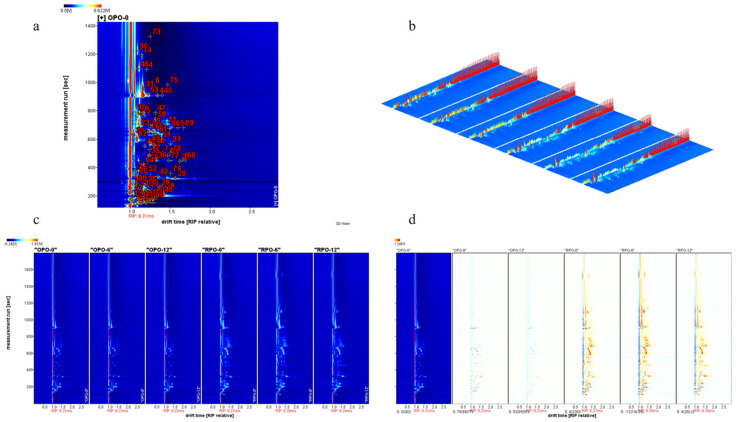
(**a**) GC-IMS spectra with detected volatile compound numbers of samples. (**b**) Three-dimensional spectrum. (**c**) Topographic plot. (**d**) Comparison of the different GC-IMS spectrum of volatile compounds.

GC-IMS is highly responsive and sensitive for the detection of trace volatile compounds, making it particularly suitable for analyzing flavor substances in foods [27]. The volatile components of OPO and RPO were analyzed using GC-IMS, and changes in their volatile profiles over the storage period were identified (Figure 3). As shown in Figure 3a, 76 volatile compounds, including alcohols, aldehydes, ketones, alkenes, pyrazines, and esters, were detected in various OPO and RPO samples. In the IMS spectrum, the horizontal axis corresponds to the ion migration time (DT), and the vertical axis corresponds to the gas chromatographic retention time (RT). The complete spectrum represents the overall volatile profiles of each peanut oil sample. Notably, numerous signal peaks were observed in the RT range of 360–1200 s and DT range of 1.0–1.8, and a red hue in the spectrum indicated a higher signal intensity. The redder the color in the spectrum, the higher the volatility signal. Figure 3b presents the 3D IMS spectra of the OPO and RPO samples, from which it is evident that the signal intensities vary significantly at different baking temperatures. Similarly, Figure 3c displays the 2D IMS plots of the peanut oil samples, confirming that the volatile signal intensities differ greatly with baking temperature. Figure 3d compares the GC-IMS spectra of OPO and RPO, using OPO-0 as a reference. In this differential plot, white denotes a volatile content equal to that of OPO-0, blue indicates a lower content, and red indicates a higher content. Compared with OPO-0 (Figure 3d), the other OPO samples exhibited slight blue regions, suggesting only minor decreases in certain volatiles during the storage period. In contrast, the RPO samples displayed prominent red peaks relative to the OPO samples, indicating that RPO contains higher concentrations of flavor compounds.

#### 3.2.2. Comparison of Fingerprints of Volatile Compounds

**Figure 4:**
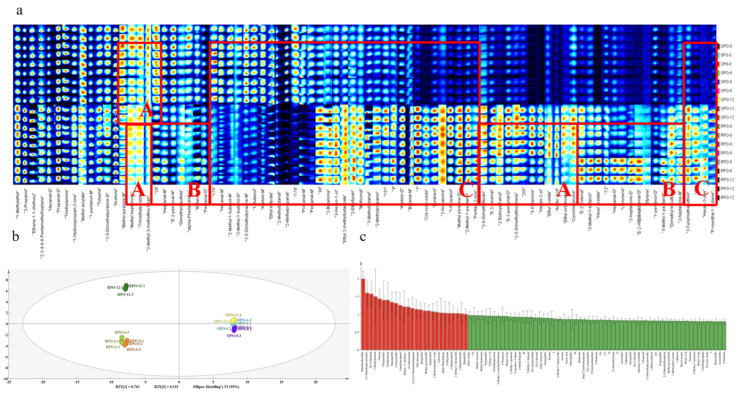
Fingerprints of the selected signal peak areas obtained from the OPO and RPO samples. Zone A represents the flavor substances, the content of which decreased with storage time. Zone B contains flavor substances whose content increased with storage time. Zone C contains flavor substances that show a significant difference in content between OPO and RPO (**a**). PCA and VIP analyses of GC-IMS spectra detected volatile compounds (**b**) PCA. (**c**) VIP values of VOCs.

The 2D and 3D GC-IMS spectra clearly reflected differences in volatile components between the OPO and RPO samples (*p* < 0.05), but provided limited insight into changes within the same sample over time. Therefore, a fingerprint plot (Figure 4a) was used to compare the volatile profiles at different storage times. Pyrazine compounds are the primary flavor constituents of peanut oil [35]. Figure 4a shows that the levels of methylpyrazine and trimethylpyrazine increased sharply in the RPO, which is consistent with the GC-MS findings. Pyrazines are end products of the Maillard reaction, and their concentrations increase at higher baking temperatures [36]. However, the levels of these pyrazines decreased over time. Figure 4a(A) illustrates that the levels of various aldehydes, ketones, and esters gradually increased during storage. For example, the levels of heptanal, butanal, pentanal, (E)-2-octenal, and (E)-2-heptenal increased during the storage period. These aldehydes are derived from amino acids in peanut oil via the Strecker degradation pathway [36]. It is worth noting that due to the influence of the concentration and properties of flavor substances, some flavor substances with affinity for cations may form dimers during ion migration [37]. Notably, heptanal was detected in both monomer (M) and dimer (D) forms, and the concentrations of both forms increased significantly in the RPO samples with time. Additionally, the levels of aldehydes, such as (E)-2-hexenal and (E)-2-pentenal, in RPO decreased over time (Figure 4a(B)), likely due to their volatility and loss during storage [36]. Figure 4a(C) indicates that the aldehyde and alcohol profiles differed significantly between RPO and OPO. During storage, peanut oxidation can generate aldehydes such as hexanal, (E)-2-heptenal, and (E)-2-octenal [38], which were found at higher levels in RPO than in OPO. Several alcohols (2-methyl-1-butanol, 3-hexen-1-ol, 2-methylpropanol, 1-propanol, 2-methyl-1-pentanol, 2-furylmethanethiol, etc.) also differed significantly between RPO and OPO, possibly due to dehydration during high-temperature baking [39].GC-MS provided better identification and quantification of pyrazines, while GC-IMS excelled at detecting highly volatile aldehydes and ketones, offering superior sensitivity for these compound classes and providing a complementary, visual fingerprint of the samples.

Table S1 indicates that GC-IMS detected 76 signal peaks. Consistent with the GC-MS analysis, the identified compounds were classified into 14 categories (Table S2): 19 aldehydes, 14 alcohols, 14 ketones, 7 esters, 5 pyrazines, 2 alkanes, 4 olefins, 3 furans, and 6 other volatile compounds. As an emerging technique, GC-IMS continues to expand its database; a few volatile components remain unidentified, accounting for minor differences between the GC-IMS and GC-MS results [40]. Notably, the qualitative analysis of pyrazine compounds differed between the two methods: GC-IMS failed to detect 2,6-dimethylpyrazine, ethylpyrazine, 2,3-dimethylpyrazine, and 2-ethyl-6-methylpyrazine. This discrepancy may be due to the fact that GC-IMS is better suited for the detection of low-molecular-weight volatile compounds [12].

#### 3.2.3. PCA and VIP Analysis

The differences in flavor compounds between the OPO and RPO samples (determined by GC-IMS) were objectively analyzed using principal component analysis (Figure 4b). PCA was used to reduce the dimensionality of the data and reveal the distribution patterns among the samples [40,41]. The OPO samples clustered closely in the PCA score plot (Figure 4b), indicating that their flavor profiles were better retained during storage. In contrast, RPO samples exhibited a more dispersed distribution: RPO-0 and RPO-6 appeared in the same quadrant, whereas RPO-12 fell into a different quadrant (*p* < 0.05). These observations are consistent with the GC-MS findings and indicate significant flavor differences between oils processed at different temperatures (OPO at 140 °C vs. RPO at 160 °C). Moreover, the disparity in flavor profiles increased with prolonged storage duration.

Variable importance in projection (VIP) analysis quantified each compound’s contribution to the PCA model [42]. Compounds with VIP values greater than 1 were identified as key volatile compounds (VOCs) that significantly contributed to the classification of peanut oils. As shown in Figure 4c, 24 VOCs (VIP > 1) are highlighted in red; these comprise five aldehydes, four alcohols, four ketones, one ester, two pyrazine heterocycles, one sulfur compound, two olefins, two alkanes, and three additional compounds. Changes in volatile levels led to differences in the flavor profiles of OPO and RPO samples.

### 3.3. Sensory Evaluation Analysis

Descriptive sensory analysis was used to evaluate the oil flavor. The predominant attributes were nutty, burnt, grassy, rancid, and fatty. The average score for each attribute is presented in Figure 2a. Six attributes differed significantly between OPO and RPO (*p* < 0.05), consistent with the GC-MS results. After one month, both oils exhibited a strong fatty odor (6.14 vs. 6.71 points), likely because of the high aldehyde content. RPO exhibited a stronger nutty flavor than OPO (9.57 vs. 8.43), likely due to the increased pyrazine formation. After 12 months of storage, changes in RPO flavor compounds were greater than those in OPO: the nutty and fatty notes of OPO decreased by only 18.62% and 16.29%, respectively, whereas those of RPO decreased by 37.93% and 19.08%, respectively. Both oils developed grassy (2.86 vs. 2.00 points) and rancid (1.71 vs. 3.71 points) notes, which were attributed to sulfides and nitriles. Pungency intensifies with longer storage periods [43,44].

### 3.4. Physical and Chemical Property Analysis

The color of peanut oil is an important factor influencing consumer choice. The color of peanut oil samples is characterized by brightness (L*), yellowness (b*), and redness (a*), with the overall color change indicated by ∆E) [45]. The L* and b* values of original peanut oil (OPO) were generally high (30.23 < L* < 38.88; 7.34 < b* < 9.82), whereas the a* and ∆E values of roasted peanut oil (RPO) were higher than those of OPO. This difference arose from the Maillard reaction between amino acids and reducing sugars at high roasting temperatures, resulting in a reddish-brown color and a significant increase in ∆E [46]. These results confirm that the roasting temperature significantly affected the peanut oil color, as evidenced by the distinct variations (*p* < 0.05) among samples pressed at different temperatures (Table 3). During storage, the oils underwent oxidation, leading to a decrease in the L* and b* values and an increase in the a* value. Notably, RPO exhibited more pronounced color changes and a higher degree of oxidation, likely due to the higher roasting temperature used for its production.

AV and PV are important parameters for evaluating the quality of vegetable oils. During production or storage, the hydrolysis of triglycerides into free fatty acids increases the AV, indicating rancidity [47]. PV reflects oil oxidation by measuring primary oxidation products. During storage, factors such as temperature fluctuations and oxygen exposure allow free radicals to attack unsaturated fatty acids, producing hydroperoxides. This significantly increases the oil’s acidity, and PV generates pungent off-odors (e.g., a sweaty or smoky odor) [48]. Throughout storage, AV and PV increased in both OPO and RPO; both remained below the FAO/WHO recommended limits (4 mg KOH/g and 5 mmol/kg, respectively; Table 4), thus meeting the edible oil standards [48]. At 6 months, AV increased by 6.94% and 9.52% in the OPO and RPO groups, respectively, whereas PV increased by 15.48% and 46.56%, respectively. By 12 months, AV increases reached 4.35% (OPO) and 6.94% (RPO), and PV increases reached 33.85% and 36.96%, respectively. RPO samples exhibited a faster rate of increase in both AV and PV compared to OPO during the storage period. 

Phytosterols and vitamin E are important antioxidant components of vegetable oils [49]. We found that the RPO samples contained significantly higher levels of vitamin E (VE) and sterols than the OPO samples (Table 4). Previous studies have similarly reported that VE and sterol contents increase with roasting temperature (120–160 °C) [48]. Both OPO and RPO exhibited only slight decreases in VE and sterol content during storage, demonstrating their good stability. During storage, the VE and sterol losses in RPO reached 4.83% and 2.69% by 6 months and 11.65% and 4.25% by 12 months, respectively. These reductions were more pronounced than those in OPO, likely due to the more extensive oxidation of unsaturated fatty acids in RPO during storage.

### 3.5. Correlation Analysis of Flavor Compounds with the Physicochemical Properties and Sensory Evaluation

**Figure 5:**
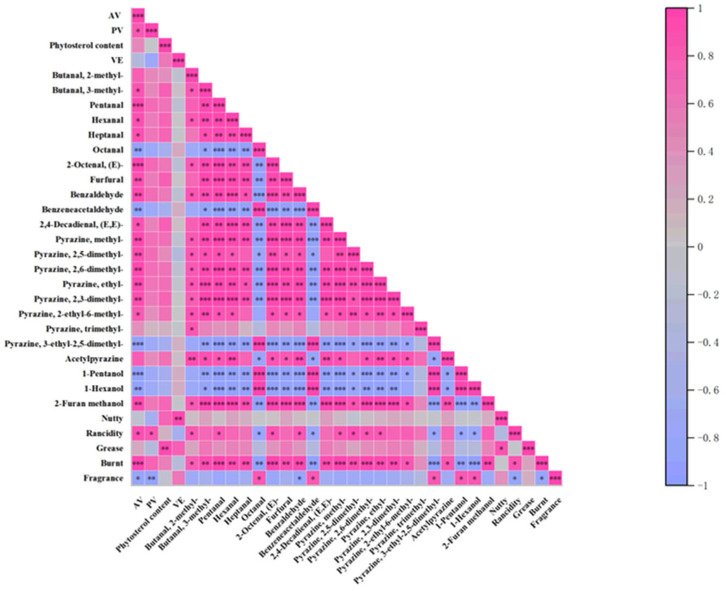
Correlation analysis heatmap of flavor compounds with the physicochemical properties and sensory evaluation(* *p* <= 0.05, ** *p* <= 0.01, *** *p* <= 0.001).

To further investigate the relationships between flavor compounds and both physicochemical properties and sensory attributes, we performed a Spearman correlation analysis on flavor compounds with an odor activity value (ROAV) > 1 (including pyrazines, alcohols, and aldehydes) and the acid value (AV), peroxide value (PV), vitamin E (VE), sterol content, and sensory scores of the samples (Figure 5). The results revealed that the key markers of lipid oxidation, hexanal, heptanal, 2-octenal, and 2,4-decadienal, which contribute to grassy, fatty, and oily off-odors [50], increased significantly with elevated AV and PV. These compounds exhibited strong negative correlations with sensory scores, indicating their major role in reducing oil acceptability. Additionally, they were strongly negatively correlated with VE content, suggesting that VE effectively inhibits lipid oxidation and thus prevents the formation of these undesirable flavor compounds. In contrast, certain pyrazines, such as methylpyrazine, 2,5-dimethylpyrazine, and trimethylpyrazine, which impart pleasant nutty, roasted, and toasted aromas, are considered core contributors to high-quality oil flavors [51]. These pyrazines exhibited strong positive correlations with sensory scores and negative correlations with AV and PV, implying that they may degrade or react with oxidation products during lipid oxidation. Other aldehydes, such as benzaldehyde (which provides a nut-like aroma [52]), can contribute positively to the flavor at appropriate concentrations. Additionally, short-chain fatty acids such as acetic acid (vinegary) and hexanoic acid (sweaty) significantly lowered the sensory scores. Therefore, in oil quality control during storage, in addition to monitoring AV and PV, it is advisable to prioritize the detection of aldehydes, such as hexanal, and characteristic pyrazines, such as trimethylpyrazine. These compounds can serve as direct and sensitive indicators of changes in oil flavor quality.

## 4. Conclusions

**Figure 6:**
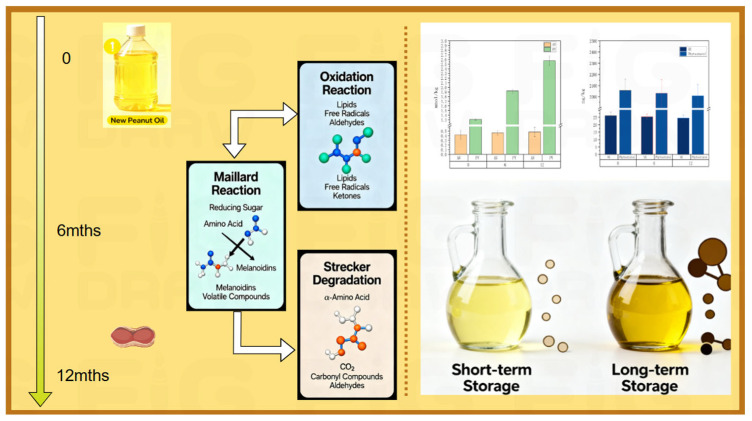
A comprehensive model illustrating the formation pathways of key flavor compounds and their correlation with the deterioration of quality parameters in hot-pressed peanut oil during storage.

GC-MS and GC-IMS were employed to characterize the flavor profiles of hot-pressed peanut oil produced at different baking temperatures and to monitor changes in these flavor compounds over 0–12 months of storage. A total of 80 volatile flavor compounds were identified by GC-MS, and 76 were detected by GC-IMS. While the overall diversity of flavor compounds was comparable between the oils, RPO contained significantly higher levels of key flavor substances and a greater number of compounds with ROAV > 1. However, extended storage led to a more rapid decline in flavor compounds in RPO, resulting in a more pronounced divergence from that of OPO. Sensory evaluation corroborated the following findings: RPO received the highest score at month 0 but developed distinct off-odors by month 12. Correlation analysis of the core flavor compounds with physicochemical and sensory data indicated that aldehydes and pyrazines are direct and sensitive indicators of changes in peanut oil flavor quality. The core findings of this study, which elucidate the intrinsic link between the evolution of flavor compounds and the deterioration of physicochemical quality during roasting and storage, are summarized in Figure 6. This schematic integrates the formation pathways of key flavor compounds (e.g., pyrazines and aldehydes) via the Maillard reaction and lipid oxidation, and correlates them with the observed changes in quality parameters during storage—namely the increase in acid value (AV) and peroxide value (PV), concurrent with the decrease in vitamin E and phytosterol contents. This model clearly illustrates the dynamic competition between the chemical reactions responsible for generating desirable flavors (e.g., nuttiness) and the oxidative degradation pathways that lead to rancidity and nutritional loss. This work not only provides a mechanistic understanding of the flavor and quality evolution in peanut oil but also establishes a theoretical foundation for the industry to concurrently optimize sensory attributes and preserve nutritional value through targeted process control and storage management. This study characterized the flavor profiles of hot-pressed peanut oil produced at different temperatures and stored for varying periods, although the underlying mechanisms driving these changes remain to be elucidated. These findings provide a theoretical foundation for optimizing the flavor profile and sensory quality of hot-pressed peanut oil. Manufacturers can select roasting temperatures based on the target flavor profile (e.g., 140 °C for nutty notes, 160 °C for roasted notes) and implement storage conditions (e.g., light avoidance, lower temperatures) to decelerate flavor degradation. Formulation strategies, such as the addition of natural antioxidants, could be developed based on our findings to inhibit the formation of undesirable oxidation-derived flavor compounds and prolong shelf life. This enhanced discussion underscores the translational potential of our research for the edible oil industry.

## Figures and Tables

**Table 1:** Criteria for sensory evaluation.

Taste	Standard for Evaluation
nutty	0–2 point: almost tasteless
rancidity	3–4 point: the taste is weak and dissipates easily
grease	5–6 point: the taste is medium and easy to detect
burnt	7–8 point: the flavor is intense for a short time
fragrance	9–10 point: the flavor is strong and lasts for a long time

**Table 2:** ROAV changes in OPO and RPO during storage period.

Name	Threshold Value	ROAV					
		OPO-0	OPO-6	OPO-12	RPO-0	RPO-6	RPO-12
Pentane	340	ND	ND	ND	0.002 ± 0.0001 ^b^	0.0025 ± 0.0003 ^c^	0.0024 ± 0.0005 ^c^
Heptane	2.70	0.07 ± 0.01 ^ab^	0.07 ± 0.00 ^a^	0.06 ± 0.00 ^a^	0.11 ± 0.01 ^c^	0.12 ± 0.01 ^c^	0.10 ± 0.02 ^bc^
Octane	8.00	0.03 ± 0.00 ^a^	0.03 ± 0.00 ^a^	0.03 ± 0.00 ^a^	0.07 ± 0.00 ^b^	0.07 ± 0.01 ^b^	0.08 ± 0.00 ^b^
Acetonitrile	22.0	0.18 ± 0.01 ^c^	0.18 ± 0.01 ^c^	0.17 ± 0.01 ^c^	0.12 ± 0.01 ^b^	0.12 ± 0.01 ^b^	ND
Acetic acid, methyl ester	5.10	0.04 ± 0.0005 ^a^	0.04 ± 0.00 ^a^	0.04 ± 0.00 ^a^	0.17 ± 0.01 ^b^	0.21 ± 0.01 ^d^	0.20 ± 0.00 ^c^
Dibutyl phthalate	0.26	0.67 ± 0.02 ^d^	0.55 ± 0.05 ^c^	0.45 ± 0.04 ^b^	ND	ND	ND
Acetamide	140.00	0.0011 ± 0.00 ^a^	0.0013 ± 0.00 ^a^	0.0014 ± 0.00 ^a^	0.0037 ± 0.00 ^b^	0.0039 ± 0.00 ^b^	0.0042 ± 0.00 ^c^
Butanal, 2-methyl-	0.140	7.35 ± 0.49 ^ab^	7.63 ± 0.11 ^ab^	6.99 ± 0.60 ^a^	8.15 ± 0.60 ^bc^	10.16 ± 0.41 ^d^	8.65 ± 0.34 ^c^
Butanal, 3-methyl-	0.0004	2464.08 ± 121.39 ^b^	2350.86 ± 177.34 ^ab^	2169.8 ± 175.17 ^a^	3069.41 ± 121.14 ^c^	3109.29 ± 113.45 ^c^	2946.55 ± 97.89 ^c^
Pentanal	0.850	0.18 ± 0.03 ^a^	0.20 ± 0.01 ^a^	0.19 ± 0.01 ^a^	0.58 ± 0.05 ^b^	0.57 ± 0.02 ^b^	0.60 ± 0.02 ^b^
Hexanal	0.001	899.64 ± 63.31 ^a^	1007.05 ± 35.83 ^a^	997.03 ± 59.06 ^a^	2679.51 ± 212.85 ^c^	2770.91 ± 207.39 ^c^	2166.10 ± 119.99 ^b^
Heptanal	0.001	235.61 ± 19.99 ^a^	275.80 ± 12.58 ^a^	271.31 ± 23.68 ^a^	706.94 ± 30.38 ^d^	521.62 ± 27.08 ^b^	611.04 ± 26.68 ^c^
Octanal	0.0009	353.99 ± 34.72 ^c^	329.22 ± 29.99 ^c^	241.71 ± 14.17 ^b^	ND	ND	ND
2-Octenal, (E)-	0.003	ND	ND	ND	102.83 ± 3.89 ^b^	118.86 ± 1.92 ^c^	119.22 ± 5.40 ^c^
Furfural	2.80	0.64 ± 0.03 ^b^	0.51 ± 0.02 ^a^	0.50 ± 0.05 ^a^	2.06 ± 0.09 ^d^	1.75 ± 0.09 ^c^	1.71 ± 0.07 ^c^
Benzaldehyde	0.085	6.26 ± 0.20 ^a^	7.44 ± 0.17 ^ab^	8.11 ± 0.49 ^b^	14.28 ± 0.91 ^c^	16.52 ± 0.90 ^d^	14.10 ± 0.97 ^c^
Benzeneacetaldehyde	0.002	154.06 ± 13.58 ^c^	148.03 ± 12.05 ^c^	112.69 ± 7.23 ^b^	ND	ND	ND
2,4-Decadienal, (E,E)-	0.002	ND	ND	ND	267.38 ± 5.97 ^d^	244.89 ± 3.82 ^c^	174.77 ± 17.98 ^b^
Disulfide, dimethyl	0.001	153.21 ± 11.90 ^bc^	161.64 ± 7.85 ^c^	143.84 ± 15.33 ^b^	ND	ND	ND
Pyrazine, methyl-	2.00	3.20 ± 0.19 ^a^	3.21 ± 0.13 ^a^	2.76 ± 0.34 ^a^	10.69 ± 0.39 ^b^	11.17 ± 1.23 ^b^	10.98 ± 1.05 ^b^
Pyrazine, 2,5-dimethyl-	0.17	57.62 ± 5.26 ^a^	56.4 ± 1.18 ^a^	49.47 ± 4.89 ^a^	66.39 ± 1.19 ^b^	75.46 ± 4.96 ^c^	78.93 ± 4.71 ^c^
Pyrazine, 2,6-dimethyl-	1.72	1.79 ± 0.14 ^a^	1.41 ± 0.15 ^a^	1.37 ± 0.06 ^a^	6.16 ± 0.16 ^b^	6.78 ± 0.43 ^c^	6.55 ± 0.42 ^bc^
Pyrazine, ethyl-	2.00	0.78 ± 0.08 ^a^	0.61 ± 0.04 ^a^	0.62 ± 0.01 ^a^	1.70 ± 0.09 ^b^	1.99 ± 0.18 ^c^	1.73 ± 0.16 ^b^
Pyrazine, 2,3-dimethyl-	0.880	0.74 ± 0.09 ^a^	0.63 ± 0.01 ^a^	0.62 ± 0.05 ^a^	1.64 ± 0.21 ^b^	1.65 ± 0.16 ^b^	1.48 ± 0.07 ^b^
Pyrazine, 2-ethyl-6-methyl-	0.040	122.47 ± 5.08 ^bc^	112.03 ± 7.26 ^ab^	96.87 ± 7.17 ^a^	137.02 ± 1.96 ^cd^	153.81 ± 19.37 ^cd^	142.24 ± 5.05 ^d^
Pyrazine, trimethyl-	0.050	48.38 ± 1.22 ^b^	45.62 ± 3.97 ^ab^	41.72 ± 2.23 ^a^	43.21 ± 0.72 ^ab^	57.44 ± 3.12 ^c^	47.89 ± 2.15 ^b^
Pyrazine, 3-ethyl-2,5-dimethyl-	0.004	668.41 ± 49.79 ^c^	540.61 ± 2.53 ^b^	555.31 ± 44.12 ^b^	ND	ND	ND
Acetylpyrazine	0.001	ND	ND	ND	2071.32 ± 25.08 ^c^	3509.16 ± 180.12 ^d^	1341.50 ± 157.61 ^b^
2-Heptanone	0.004	ND	ND	ND	118.63 ± 6.32 ^c^	92.25 ± 4.34 ^b^	87.07 ± 6.42 ^b^
2-Hydroxy-3-pentanone	2.501	0.09 ± 0.01 ^c^	0.07 ± 0.00 ^b^	0.07 ± 0.01 ^b^	ND	ND	ND
1,2-Cyclopentanedione, 3-methyl-	0.026	10.21 ± 1.23 ^a^	9.04 ± 0.48 ^a^	8.90 ± 0.71 ^a^	34.04 ± 0.51 ^b^	38.42 ± 1.91 ^c^	34.11 ± 1.23 ^b^
Furan, 2-pentyl-	0.019	ND	ND	ND	19.83 ± 1.35 ^b^	29.92 ± 2.19 ^c^	33.67 ± 0.19 ^d^
Pyridine	0.320	ND	ND	ND	2.88 ± 0.10 ^c^	3.06 ± 0.10 ^c^	2.24 ± 0.34 ^b^
Pyridine, 2-propyl-	0.011	ND	ND	ND	34.93 ± 2.03 ^b^	40.84 ± 1.56 ^c^	47.50 ± 2.71 ^d^
1-Pentanol	0.153	0.88 ± 0.07 ^c^	0.86 ± 0.03 ^c^	0.69 ± 0.07 ^b^	ND	ND	ND
1-Hexanol	0.034	6.77 ± 0.30 ^c^	5.05 ± 0.18 ^b^	5.06 ± 0.35 ^b^	ND	ND	ND
2-Furan methanol	2.800	0.78 ± 0.04 ^a^	0.60 ± 0.04 ^a^	0.58 ± 0.03 ^a^	3.30 ± 0.08 ^c^	3.50 ± 0.20 ^c^	3.02 ± 0.16 ^b^
Acetic acid	0.013	338.38 ± 19.95 ^a^	334.46 ± 16.59 ^a^	330.63 ± 3.85 ^a^	2042.87 ± 93.93 ^b^	2847.40 ± 154.35 ^d^	2242.50 ± 97.75 ^c^
Hexanoic acid	0.005	49.09 ± 3.60 ^a^	50.60 ± 6.72 ^a^	52.48 ± 5.45 ^a^	379.27 ± 10.12 ^c^	302.01 ± 10.98 ^b^	313.46 ± 4.53 ^b^
Nonanoic acid	0.120	4.09 ± 0.47 ^c^	3.85 ± 0.15 ^bc^	3.14 ± 0.24 ^a^	3.48 ± 0.13 ^ab^	3.60 ± 0.24 ^abc^	3.95 ± 0.09 ^bc^
Pentanoic acid	0.0002	ND	ND	ND	867.17 ± 49.82 ^bc^	806.80 ± 23.58 ^b^	896.69 ± 68.51 ^c^
2-Methoxy-4-vinylphenol	0.003	201.02 ± 17.55 ^a^	176.53 ± 9.63 ^a^	163.11 ± 23.34 ^a^	447.28 ± 54.54 ^c^	386.44 ± 23.9 ^b^	398.70 ± 17.97 ^bc^

ND: not detected. Values represent the means ± standard deviation; *n* = 3. Different letters (a, b, c, d) in each column indicate significant differences (*p* < 0.05).

**Table 3:** Color change in peanut oil during storage.

Name	L	a	b	∆E
OPO-0	38.88 ± 1.05 ^a^	1.65 ± 0.15 ^b^	9.82 ± 1.50 ^e^	6.24 ± 2.12 ^f^
OPO-6	35.30 ± 2.35 ^b^	2.03 ± 0.09 ^c^	8.67 ± 1.13 ^e^	6.77 ± 1.99 ^d^
OPO-12	30.23 ± 1.89 ^b^	4.26 ± 0.12 ^cd^	7.34 ± 2.23 ^d^	8.96 ± 0.77 ^b^
RPO-0	24.14 ± 2.18 ^b^	6.33 ± 0.05 ^d^	14.17 ± 2.87 ^c^	8.47 ± 1.50 ^e^
RPO-6	20.94 ± 1.99 ^c^	7.20 ± 0.02 ^e^	13.01 ± 2.12 ^b^	10.40 ± 0.99 ^c^
RPO-12	15.87 ± 1.45 ^d^	10.35 ± 0.56 ^a^	11.68 ± 3.45 ^a^	14.20 ± 3.12 ^a^

Different letter superscripts within the same column indicate significant differences (*p* < 0.05).

**Table 4:** Analysis of the physical and chemical properties of OPO and RPO during storage.

Sample	AV mg KOH/g	PV mmol/kg	Phytosterol Content mg/kg	VE mg/kg
OPO-0	0.42 ± 0.10 ^a^	1.31 ± 0.02 ^a^	2057 ± 100.00 ^bc^	26.13 ± 2.50 ^e^
OPO-6	0.46 ± 0.05 ^a^	1.92 ± 0.05 ^b^	2032 ± 121.00 ^b^	25.41 ± 1.80 ^d^
OPO-12	0.48 ± 0.10 ^b^	2.57 ± 0.10 ^d^	2009 ± 102.00 ^a^	24.46 ± 2.00 ^b^
RPO-0	0.72 ± 0.15 ^c^	2.39 ± 0.12 ^c^	2134 ± 120.52 ^d^	26.52 ± 3.12 ^f^
RPO-6	0.77 ± 0.08 ^d^	2.76 ± 0.06 ^e^	2078 ± 210.00 ^cd^	25.24 ± 2.24 ^c^
RPO-12	0.84 ± 0.03 ^e^	3.78 ± 0.06 ^f^	2047 ± 103.00 ^bc^	23.43 ± 1.50 ^a^

Different letter superscripts within the same column indicate significant differences (*p* < 0.05).

## Data Availability

The data used to support the findings of this study can be made available by the corresponding author upon request.

## Supplementary Materials

The following supporting information can be downloaded at: https://www.mdpi.com/article/10.3390/foods14223945/s1, Table S1: Concentration changes of volatile compounds in peanut oils during storage period; Table S2: Determination of volatile components by GC-MS.
