# Supplementary material for: Flavor Evolution and Quality Changes in Hot-Pressed Peanut Oil: Impact of Roasting Temperature and Storage Time
Source: Foods. 2025 Nov 18;14(22):3945. doi: 10.3390/foods14223945 (PMC12651882; doi:10.3390/foods14223945)
Supplement: Supplementary file 1 [file foods-14-03945-s001.zip › highlight.pdf]

Highlights:

1. **GC-MS and GC-IMS identified 80 and 86 volatile compounds in peanut oils, respectively.**
2. **Extended storage led to a more rapid decline in the flavor compounds of RPO.**
3. **Sensory evaluation showed that RPO had the highest initial score but developed off-odors by 12 months.**
4. **More pronounced attenuation of flavor compounds in peanut oil roasted at 160 ° C during storage**
5. **Correlation analysis revealed that changes in aldehydes and pyrazines are closely associated with lipid oxidation and sensory quality.**
