# Supplementary material for: Flavor Evolution and Quality Changes in Hot-Pressed Peanut Oil: Impact of Roasting Temperature and Storage Time
Source: Foods. 2025 Nov 18;14(22):3945. doi: 10.3390/foods14223945 (PMC12651882; doi:10.3390/foods14223945)
Supplement: Supplementary file 1 [file foods-14-03945-s001.zip › supplementary tables.pdf]

Table S1. Concentration changes of volatile compounds in peanut oils during storage period.

No.	Category	Name	Cas	Concentration（mg/kg）					
				OPO-0	OPO-6	OPO-12	RPO-0	RPO-6	RPO-12
1.	Alkane	Pentane	109-66-0	ND	ND	ND	0.68±0.04^{b}	0.85±0.10^{a}	0.81±0.08^{a}
2.		Heptane	142-82-5	0.20±0.04^{c}	0.19±0.01^{c}	0.17±0.01^{c}	0.31±0.03^{b}	0.32±0.03^{b}	0.28±0.06^{b}
3.		Octane	111-65-9	0.23±0.02^{b}	0.22±0.01^{b}	0.21±0.01^{b}	0.58±0.04^{a}	0.59±0.06^{a}	0.64±0.04^{a}
4.		Cyclopropane, pentyl-	2511-91-3	0.80±0.07^{c}	0.78±0.05^{c}	0.66±0.05^{c}	0.99±0.04^{b}	1.20±0.040^{a}	1.22±0.15^{a}
5.	Nitrile	Acetonitrile	27522.00	3.91±0.40^{a}	3.93±0.17^{a}	3.73±0.32^{a}	2.72±0.19^{b}	2.59±0.16^{b}	ND
6.	Esters	Acetic acid, methyl ester	79-20-9	0.21±0.02^{c}	0.22±0.01^{c}	0.22±0.02^{c}	0.86±0.08^{b}	1.09±0.09^{a}	1.02±0.02^{a}
7.		Butyrolactone	96-48-0	0.43±0.03^{a}	0.40±0.03^{a}	0.40±0.04^{a}	ND	ND	ND
8.		2(3H)-Furanone, dihydro-3-hydroxy-4,4-dimethyl-, (±)-	79-50-5	0.27±0.02^{bc}	0.25±0.04^{c}	0.25±0.04^{bc}	0.36±0.04^{a}	0.38±0.06^{a}	0.33±0.02^{ab}
9.		Dibutyl phthalate	84-74-2	0.17±0.01^{a}	0.14±0.02^{b}	0.12±0.01^{c}	ND	ND	ND
10.	Amines	Hydroxylamine	7803-49-8	0.30±0.06^{a}	0.31±0.01^{a}	0.31±0.04^{a}	ND	ND	ND
11.		(2-Aziridinylethyl)amine	4025-37-0	0.13±0.01^{a}	0.14±0.00^{a}	0.12±0.01^{a}	ND	ND	ND

12.		2-OXOPROPIONAMIDE	631-66-3	0.15±0.01[c]	0.16±0.01[c]	0.16±0.02[c]	0.61±0.02[b]	0.67±0.04[a]	0.62±0.04[b]
13.		Acetamide	60-35-5	0.17±0.02[c]	0.17±0.01[c]	0.17±0.01[c]	0.52±0.03[b]	0.55±0.04[ab]	0.59±0.02[a]
14.		Formamide, N-phenyl-	103-70-8	0.39±0.09[a]	0.36±0.02[a]	0.27±0.02[b]	ND	ND	ND
15.	Aldehydes	Butanal, 2-methyl-	96-17-3	1.03±0.08[cd]	1.07±0.02[bcd]	0.98±0.10[d]	1.14±0.1[bc]	1.42±0.07[a]	1.21±0.06[b]
16.		Butanal, 3-methyl-	590-86-3	0.86±0.05[b]	0.82±0.08[b]	0.76±0.08[b]	1.07±0.05[a]	1.09±0.05[a]	1.03±0.04[a]
17.		Pentanal	110-62-3	0.15±0.03[b]	0.17±0.01[b]	0.16±0.01[b]	0.49±0.05[a]	0.49±0.02[a]	0.51±0.02[a]
18.		Hexanal	66-25-1	1.26±0.11[c]	1.41±0.06[c]	1.40±0.10[c]	3.75±0.36[a]	3.88±0.36[a]	3.03±0.21[b]
19.		2-methyl-2-butenal	497-03-0	0.13±0.01[a]	0.12±0.01[a]	0.10±0.01[b]	ND	ND	ND
20.		Heptanal	111-71-7	0.21±0.02[d]	0.25±0.01[d]	0.24±0.03[d]	0.64±0.03[a]	0.47±0.03[c]	0.55±0.03[b]
21.		Octanal	124-13-0	0.31±0.04[a]	0.29±0.03[a]	0.21±0.02[b]	ND	ND	ND
22.		2-Octenal, (E)-	2548-87-0	ND	ND	ND	0.28±0.01[b]	0.32±0.01[a]	0.32±0.02[a]
23.		Furfural	98-01-1	1.79±0.09[c]	1.42±0.05[c]	1.41±0.17[c]	5.77±0.29[a]	4.89±0.32[b]	4.78±0.22[b]
24.		Benzaldehyde	100-52-7	0.53±0.02[d]	0.63±0.02[cd]	0.69±0.05[c]	1.21±0.09[b]	1.4±0.09[a]	1.20±0.10[b]
25.		Pyrrole-2-carboxaldehyde	1003-29-8	0.55±0.03[a]	0.52±0.01[a]	0.49±0.04[b]	ND	ND	ND

26.		1H-Pyrrole-2-carboxaldehyde, 1-methyl-	1192-58-1	0.22±0.00[d]	0.20±0.02[d]	0.18±0.01[d]	0.89±0.03[c]	1.29±0.2[b]	2.00±0.09[a]
27.		2-Furancarboxaldehyde, 5-methyl-	620-02-0	0.31±0.01[b]	0.27±0.02[b]	0.28±0.02[b]	5±0.62[a]	5.05±0.11[a]	4.91±0.33[a]
28.		Benzeneacetaldehyde	122-78-1	0.26±0.03[a]	0.25±0.03[a]	0.19±0.02[b]	ND	ND	ND
29.		2,4-Decadienal, (E,E)-	25152-84-5	ND	ND	ND	0.61±0.02[b]	0.56±0.01[c]	0.40±0.05[d]
30.		1H-Pyrrole-2-carboxaldehyde	1003-29-8	ND	ND	ND	5.94±0.17[b]	6.29±0.36[ab]	6.49±0.40[a]
31.	Pyrrole	2-Acetyl pyrrole	1072-83-9	0.65±0.05[c]	0.55±0.01[c]	0.55±0.07[c]	0.97±0.03[b]	1.10±0.04[a]	0.9±0.08[b]
32.	Sulphide	Disulfide, dimethyl	624-92-0	0.15±0.01[a]	0.16±0.01[a]	0.14±0.02[a]	ND	ND	ND
33.	Prazine	Pyrazine	290-37-9	0.16±0.02[c]	0.17±0.01[c]	0.14±0.01[c]	1.06±0.06[b]	1.22±0.04[a]	1.12±0.08[b]
34.		Pyrazine, methyl-	109-08-0	6.40±0.47[b]	6.42±0.32[b]	5.52±0.83[b]	21.38±0.95[a]	22.34±3.01[a]	21.96±2.57[a]
35.		Pyrazine, 2,5-dimethyl-	123-32-0	9.8±1.10[cd]	9.59±0.25[cd]	8.41±1.02[d]	11.29±0.25[bc]	12.83±1.03[ab]	13.42±0.98[a]
36.		Pyrazine, 2,6-dimethyl-	108-50-9	3.08±0.28[b]	2.42±0.32[b]	2.35±0.13[b]	10.60±0.33[a]	11.66±0.91[a]	11.27±0.89[a]
37.		Pyrazine, ethyl-	13925-00-3	1.56±0.19[c]	1.22±0.09[c]	1.24±0.03[c]	3.40±0.23[b]	3.98±0.44[a]	3.46±0.40[ab]
38.		Pyrazine, 2,3-dimethyl-	5910-89-4	0.65±0.09[b]	0.55±0.01[b]	0.55±0.05[b]	1.44±0.22[a]	1.45±0.17[a]	1.31±0.08[a]

39.		Pyrazine, 2-ethyl-6-methyl-	13925-03-6	$4.90±0.25^{bc}$	$4.48±0.36^{cd}$	$3.87±0.35^{d}$	$5.48±0.10^{ab}$	$6.15±0.95^{a}$	$5.69±0.25^{ab}$
40.		Pyrazine, trimethyl-	14667-55-1	$2.42±0.07^{b}$	$2.28±0.24^{bc}$	$2.09±0.14^{c}$	$2.16±0.04^{bc}$	$2.87±0.19^{a}$	$2.39±0.13^{b}$
41.		Pyrazine, 3-ethyl-2,5-dimethyl-	13360-65-1	$2.41±0.22^{a}$	$1.95±0.01^{b}$	$2.00±0.19^{b}$	ND	ND	ND
42.		Pyrazine, 2-ethenyl-6-methyl-	13925-09-2	$0.27±0.01^{b}$	$0.20±0.01^{c}$	$0.21±0.01^{c}$	$0.43±0.02^{a}$	$0.44±0.03^{a}$	$0.43±0.02^{a}$
43.		Pyrazine, 3,5-diethyl-2-methyl-	18138-05-1	$0.25±0.02^{a}$	$0.27±0.02^{a}$	$0.26±0.02^{a}$	ND	ND	ND
44.		Pyrazine, 2-methyl-6-(1-propenyl)-, (E)-	55138-66-4	$0.24±0.03^{a}$	$0.21±0.01^{ab}$	$0.20±0.02^{b}$	ND	ND	ND
45.		5H-5-Methyl-6,7-dihydrocyclopentapyrazinedihydrocyclopentapyrazine	23747-48-0	$0.20±0.02^{a}$	$0.16±0.01^{b}$	$0.16±0.01^{b}$	ND	ND	ND
46.		Pyrazine, (1-methylethenyl)-	38713-41-6	$0.17±0.00^{a}$	$0.16±0.03^{a}$	$0.13±0.00^{b}$	ND	ND	ND
47.		2-acetyl-6-methyl pyrazine	22047-26-3	$0.29±0.04^{b}$	$0.22±0.03^{b}$	$0.21±0.01^{b}$	$2.40±0.03^{a}$	$2.54±0.38^{a}$	$2.24±0.24^{a}$
48.		Acetylpyrazine	22047-25-2	ND	ND	ND	$0.83±0.01^{b}$	$1.40±0.09^{a}$	$0.54±0.08^{c}$
49.		Pyrazine, (1-methylethenyl)-	38713-41-6	ND	ND	ND	$0.38±0.04^{b}$	$0.43±0.03^{a}$	$0.47±0.01^{a}$
50.	Ketones	2-Heptanone	110-43-0	ND	ND	ND	$0.42±0.03^{a}$	$0.32±0.02^{b}$	$0.30±0.03^{b}$
51.		Acetoin	513-86-0	$0.27±0.03^{b}$	$0.24±0.02^{b}$	$0.22±0.02^{b}$	$0.48±0.04^{a}$	$0.53±0.03^{a}$	$0.50±0.04^{a}$

52.		2-Propanone, 1-hydroxy-	116-09-6	0.36±0.04[b]	0.30±0.02[b]	0.28±0.02[b]	2.30±0.11[a]	2.31±0.19[a]	2.36±0.16[a]
53.		2-Hydroxy-3-pentanone	5704-20-1	0.23±0.03[a]	0.18±0.01[b]	0.18±0.02[b]	ND	ND	ND
54.		2-Propanone, 1-(acetyloxy)-	592-20-1	0.37±0.04[b]	0.38±0.04[b]	0.38±0.04[b]	1.85±0.21[a]	1.68±0.11[a]	1.64±0.20[a]
55.		2(3H)-Furanone, dihydro-4-methyl-	1679-49-8	0.28±0.01[a]	0.23±0.01[b]	0.24±0.02[b]	ND	ND	ND
56.		2(5H)-Furanone	497-23-4	0.30±0.02[c]	0.25±0.02[c]	0.25±0.03[c]	0.88±0.04[b]	0.91±0.06[ab]	0.96±0.05[a]
57.		1,2-Cyclopentanedione, 3-methyl-	765-70-8	0.27±0.04[c]	0.24±0.02[c]	0.23±0.02[c]	0.88±0.02[b]	1.00±0.06[a]	0.89±0.04[b]
58.	Furans	Furan, 2-pentyl-	3777-69-3	ND	ND	ND	0.38±0.03[c]	0.57±0.05[b]	0.64±0.00[a]
59.		Ethanone, 1-(2-furanyl)-	1192-62-7	ND	ND	ND	0.97±0.07[b]	1.11±0.07[a]	0.97±0.05[b]
60.	Pyridine	Pyridine	110-86-1	ND	ND	ND	0.92±0.04[a]	0.98±0.04[a]	0.72±0.13[b]
61.		Pyridine, 2-propyl-	622-39-9	ND	ND	ND	0.38±0.03[c]	0.45±0.02[b]	0.52±0.04[a]
62.		Pyridine, 3-methoxy-	7295-76-3	0.16±0.01[a]	0.16±0.01[a]	0.18±0.01[a]	ND	ND	ND
63.	Alcohols	3-Pentanol, 2-methyl-	565-67-3	0.27±0.03[a]	0.24±0.01[b]	0.23±0.02[b]	ND	ND	ND
64.		1-Pentanol	71-41-0	0.13±0.01[a]	0.13±0.00[a]	0.11±0.01[b]	ND	ND	ND

65.		1-Hexanol	111-27-3	0.23±0.01[a]	0.17±0.01[b]	0.17±0.01[b]	ND	ND	ND
66.		2-Furan methanol	98-00-0	2.19±0.13[c]	1.68±0.12[c]	1.62±0.11[c]	9.23±0.26[ab]	9.79±0.70[a]	8.46±0.55[b]
67.		2-Furanmethanol, 5-methyl-	3857-25-8	ND	ND	ND	0.75±0.07[a]	0.49±0.03[b]	ND
68.		Glycerin	56-81-5	1.61±0.19[a]	1.36±0.15[b]	1.02±0.10[c]	1.48±0.10[ab]	1.25±0.13[bc]	ND
69.	Acids	Acetic acid	64-19-7	4.40±0.32[c]	4.35±0.26[c]	4.30±0.06[c]	26.56±1.50[b]	37.02±2.46[a]	29.15±1.56[b]
70.		2-Butenoic acid, 3-methyl-	541-47-9	0.13±0.01[d]	0.12±0.00[d]	0.11±0.01[d]	2.04±0.03[a]	1.55±0.12[b]	0.86±0.07[c]
71.		Pentanoic acid	105-43-1	ND	ND	ND	0.55±0.01[b]	0.62±0.05[a]	0.66±0.04[a]
72.		CH2=C(CH3)CH2COOH	1617-31-8	ND	ND	ND	1.33±0.02[b]	1.66±0.10[a]	1.34±0.07[b]
73.		Hexanoic acid	142-62-1	0.24±0.02[c]	0.24±0.04[c]	0.25±0.03[c]	1.82±0.06[a]	1.45±0.06[b]	1.5ND.03[b]
74.		Nonanoic acid	112-05-0	0.49±0.07[a]	0.46±0.02[c]	0.38±0.03[b]	0.42±0.02[ab]	0.43±0.03[ab]	0.47±0.01[b]
75.		Pentanoic acid	109-52-4	ND	ND	ND	0.14±0.01[ab]	0.13±0.00[b]	0.18±0.00[a]
76.	Phenols	Oxime-, methoxy-phenyl-	150-76-5	0.19±0.02[a]	0.19±0.02[a]	0.19±0.01[a]	ND	ND	ND
77.		Maltol	118-71-8	0.67±0.09[b]	0.54±0.01[bc]	0.49±0.05[c]	0.88±0.06[a]	0.99±0.08[a]	0.96±0.10[a]
78.		Phenol, 2-methoxy-	32994	ND	ND	ND	0.52±0.02[a]	0.53±0.07[a]	0.29±0.02[b]

79.	2-Methoxy-4-vinylphenol	7786-61-0	0.56±0.06[b]	0.49±0.03[b]	0.46±0.08[b]	1.25±0.19[a]	1.08±0.08[a]	1.12±0.06[a]
80.	4-Vinylphenol	2628-17-3	3.23±0.13[c]	2.91±0.08[c]	2.80±0.31[c]	6.77±0.11[a]	6.56±0.50[a]	5.64±0.50[b]

2

Table S2. Determination of volatile components by GC-IMS.

No.	Compound	CAS	Formula	NM	RI	RT	DT(RIPrel)	Signal intensity					
								OPO-0	OPO-6	OPO-6	RPO-0	RPO-6	RPO-12
1.	Propanal(M)	C123386	C3H6O	58.1	795.4	148.804	1.0482	618.72±5.10^{d}	695.54±11.01^{f}	661.36±14.58^{e}	90.37±3.65^{a}	208.54±1.61^{b}	227.24±3.58^{c}
2.	Propanal(D)	C123386	C3H6O	58.1	794.2	148.453	1.145	2885.36±11.45^{b}	2801.26±12.04^{a}	2842.27±5.94^{ab}	3686.55±88.14^{e}	3297.1±12.28^{c}	3571.32±46.92^{d}
3.	2-methylpropanal	C78842	C4H8O	72.1	808.3	152.68	1.2838	1442.23±8.04^{e}	1443.75±10.78^{e}	1415.03±11.69^{d}	292.02±2.79^{a}	380.49±8.93^{b}	427.38±4.96^{c}
4.	Butanal(M)	C123728	C4H8O	72.1	867.6	173.431	1.1056	1174.2±5.66^{a}	1273±7.26^{b}	1190.57±32.22^{a}	1486.26±23.98^{c}	1772.08±34.23^{e}	1694.16±15.74^{d}
5.	Butanal(D)	C123728	C4H8O	72.1	872.8	175.509	1.2815	884.17±5.09^{c}	974.07±13.08^{d}	960.13±33.52^{d}	558.47±10.85^{a}	637.32±18.8^{b}	977.98±17.72^{d}
6.	2-Methylbutanal	C96173	C5H10O	86.1	945.9	209.938	1.4057	11304.95±51.03^{d}	11245.86±175.95^{d}	10588.25±18.96^{c}	7703.4±99.02^{a}	7765.72±183.47^{a}	8151.46±80.76^{b}
7.	Pentanal(M)	C110623	C5H10O	86.1	996.5	240.764	1.1816	1113.28±14.35^{f}	976.62±7.49^{d}	1001.13±6.9^{e}	421.98±7.30^{a}	450.51±4.85^{b}	547.03±0.94^{c}
8.	Pentanal(D)	C110623	C5H10O	86.1	991.9	237.681	1.4277	1805.93±29.36^{a}	2109.11±35.75^{b}	2132.15±26.55^{b}	2915.8±13.68^{c}	3155.38±28.22^{d}	4057.82±34.07^{e}
9.	Hexanal(M)	C66251	C6H12O	100.2	1091.5	319.208	1.2575	2526.19±6.35^{d}	2567.51±9.33^{d}	2579.74±14.69^{d}	1220.36±47.78^{b}	1140.5±16.16^{a}	1313.87±46.98^{c}
10.	Hexanal(D)	C66251	C6H12O	100.2	1095.3	323.102	1.5688	3938.78±13.02^{a}	4330.56±38.91^{b}	4562.24±55.19^{c}	7196.24±130.19^{d}	7430.17±46.83^{e}	8552.02±89.08^{f}
11.	Heptanal(M)	C111717	C7H14O	114.2	1197.6	452.664	1.3278	1361.29±14.44^{c}	1438.91±10.42^{d}	1518.27±35.92^{e}	1116.72±32.30^{a}	1264.62±10.09^{b}	1688.98±43.40^{f}

12.	Heptanal(D)	C111717	C7H14O	114.2	1198.4	453.841	1.693	197.35±9.64^{a}	219.46±6.25^{b}	213.69±6.13^{ab}	255.31±8.92^{c}	339.64±14.18^{d}	610.23±7.15^{e}
13.	(E)-2-Hexenal	C6728263	C6H10O	98.1	1254.3	552.104	1.1832	484.83±25.32^{a}	615.24±7.57^{b}	655.14±19.23^{b}	1086.93±69.19^{c}	1430.25±21.26^{d}	1392.57±50.28^{d}
14.	(E)-2-Octenal	C2548870	C8H14O	126.2	1393.3	913.834	1.3375	838.3±9.18^{b}	648.95±25.39^{a}	628.25±13.53^{a}	2485.58±122.99^{d}	2257.92±78.62^{c}	2976.07±20.44^{e}
15.	Methional	C3268493	C4H8OS	104.2	1474	1224.922	1.0908	1417.88±54.82^{b}	1337.62±39.22^{b}	1225.25±39.58^{a}	2023.21±62.47^{c}	2038.51±36.47^{c}	2061.27±54.92^{c}
16.	3-Methylbutanal	C590863	C5H10O	86.1	892.9	183.984	1.1665	382.25±7.50^{b}	362.24±7.16^{a}	375.82±7.33^{b}	427.57±1.20^{c}	727.28±7.15^{e}	558.04±4.15^{d}
17.	(E)-2-Heptenal	C18829555	C7H12O	112.2	1312.4	680.95	1.6699	151.65±12.20^{a}	188.85±4.76^{b}	211.89±8.70^{bc}	194.76±21.22^{b}	233.03±7.51^{c}	237.12±14.19^{c}
18.	(E)-2-Pentenal(M)	C1576870	C5H8O	84.1	1112.2	340.847	1.1026	1098.91±3.07^{e}	1007.67±3.82^{d}	995.86±6.72^{d}	793.84±19.75^{c}	740.17±1.91^{b}	565.25±0.96^{a}
19.	(E)-2-Pentenal(D)	C1576870	C5H8O	84.1	1109.4	337.813	1.3583	871±5.94^{c}	664.36±28.93^{a}	733.01±8.40^{b}	1995.41±35.94^{f}	1870.29±8.38^{e}	1340.11±11.25^{d}
20.	2-Propanol	C67630	C3H8O	60.1	906	189.882	1.2454	5003.45±32.01^{c}	4773.48±17.20^{a}	4899.17±33.39^{b}	8711.81±76.99^{e}	8855.49±22.01^{f}	8615.21±14.85^{d}
21.	1-Propanol	C71238	C3H8O	60.1	1021.3	258.321	1.1103	492.38±8.38^{a}	518.64±7.13^{a}	498.56±9.86^{a}	2125.73±16.78^{d}	2028.12±32.37^{c}	1528.16±16.56^{b}
22.	2-Methylpropanol	C78831	C4H10O	74.1	1043.1	275.379	1.1652	121.84±3.03^{b}	107.51±9.33^{a}	125.21±1.68^{b}	193.94±4.17^{d}	163.19±4.1^{c}	129.03±2.11^{b}
23.	1-Butanol	C71363	C4H10O	74.1	1175.6	419.91	1.1759	569.4±13.47^{d}	526.19±9.85^{c}	531.57±13.39^{c}	281.09±10.77^{b}	242.37±9.38^{a}	230.39±6.08^{a}
24.	2-Methyl-1-	C137326	C5H12O	88.1	1218.	486.139	1.237	1379.87±5.6	1253.62±10.	1223.01±15	735.46±75.4	684.39±53.9	722.34±14.5

	butanol(M)				2			4^{c}	59^{b}	.72^{b}	0^{a}	5^{a}	0^{a}
25.	2-Methyl-1-butanol(D)	C137326	C5H12O	88.1	1218	485.723	1.4755	385.16±13.21^{e}	360.08±11.24^{d}	318.71±4.44^{c}	205.57±8.95^{b}	155.45±16.69^{a}	186.23±12.81^{b}
26.	3-Methyl-1-butanol	C123513	C5H12O	88.1	1196.1	450.309	1.4871	175.07±1.21^{a}	213.91±8.93^{b}	210.61±13.24^{b}	327.14±19.05^{c}	384.34±14.58^{d}	538.58±8.26^{e}
27.	1-Pentanol(M)	C71410	C5H12O	88.1	1264.8	573.22	1.2552	3158.59±16.89^{b}	2993.56±37.35^{a}	2955.55±14.20^{a}	3448.25±22.83^{e}	3222.82±5.45^{c}	3279.70±34.79^{d}
28.	1-Pentanol(D)	C71410	C5H12O	88.1	1261.9	567.224	1.506	632.71±4.82^{c}	546.29±8.96^{a}	548.21±5.73^{a}	534.33±12.93^{a}	601.62±7.10^{b}	901.21±29.52^{d}
29.	3-Methy-1-pentanol	C589355	C6H14O	102.2	1310.7	678.899	1.6006	232.00±24.39^{a}	344.70±2.72^{b}	317.87±6.03^{b}	851.16±17.84^{c}	1341.79±29.62^{d}	2023.32±25.92^{e}
30.	Hexan-2-ol	C626937	C6H14O	102.2	1224.1	494.345	1.2754	229.68±9.92^{a}	240.93±8.55^{a}	210.01±9.63^{a}	1975.07±29.88^{c}	2204.20±51.27^{b}	1829.06±26.25^{d}
31.	2-Methyl-1-pentanol	C105306	C6H14O	102.2	1303.1	658.383	1.2883	3099.56±58.83^{c}	2598.47±73.37^{b}	2436.33±36.21^{a}	7811.94±58.26^{e}	7649.04±86.80^{d}	7916.25±65.53^{e}
32.	3-Hexen-1-ol	C928961	C6H12O	100.2	1393.5	914.303	1.2284	2078.99±50.87^{ab}	2153.09±63.64^{b}	2008.11±63.91^{a}	2894.26±48.86^{c}	3659.10±38.51^{d}	3671.09±17.17^{d}
33.	2-Furylmethanethiol	C98022	C5H6OS	114.2	1445.4	1104.214	1.1093	1667.12±41.98^{b}	1690.56±43.73^{b}	1415.63±56.05^{a}	4003.92±111.39^{d}	3375.2±92.00^{c}	3893.33±19.13^{d}
34.	Acetone	C67641	C3H6O	58.1	828.6	159.219	1.1144	5992.07±12.66^{c}	5669.39±19.20^{a}	5813.97±42.55^{b}	7581.27±59.25^{f}	6938.43±23.38^{d}	7047.57±32.92^{e}
35.	2-Butanone	C78933	C4H8O	72.1	884.5	180.369	1.2502	356.44±6.06^{b}	326.98±1.87^{a}	328.74±20.92^{a}	580.15±8.75^{c}	659.57±3.87^{d}	598.66±16.08^{c}
36.	Furaneol	C365877	C6H8O3	128.1	1056.	286.976	1.1995	290.74±2.57	234.65±5.06^{a}	252.37±7.5	804.31±20.5	743.46±13.6	686.79±22.4

		3			9			^{b}		1^{a}	5^{e}	0^{d}	9^{c}
37.	Pentan-2-one	C107879	C5H10O	86.1	1030.5	265.37	1.3678	114.47±7.57^{a}	131.39±4.93^{b}	128.75±7.50^{b}	242.46±8.81^{c}	371.94±4.84^{d}	391.07±5.69^{e}
38.	2-Heptanone(M)	C110430	C7H14O	114.2	1198	453.187	1.2579	721.95±13.38^{a}	676.06±18.55^{a}	659.92±16.18^{a}	868.56±120.20^{b}	1061.16±91.53^{c}	1296.05±19.47^{d}
39.	2-Heptanone(D)	C110430	C7H14O	114.2	1192.8	445.208	1.6257	93.11±4.66^{a}	113.82±3.00^{b}	115.12±5.74^{b}	210.38±1.56^{c}	355.39±21.32^{d}	711.70±3.93^{e}
40.	Acetoin(M)	C513860	C4H8O2	88.1	1290.8	629.472	1.0539	3127.6±31.35^{c}	3122.37±22.74^{c}	3171.75±15.57^{c}	1612.63±42.20^{a}	1627.72±10.86^{a}	1703.01±26.89^{b}
41.	Acetoin(D)	C513860	C4H8O2	88.1	1293.2	634.99	1.3387	7638.65±119.18^{b}	6827.45±349.26^{a}	6378.74±191.53^{a}	14557.15±316.01^{d}	12597.13±182.92^{c}	12353±98.07^{c}
42.	1-Hydroxypropan-2-one	C116096	C3H6O2	74.1	1302.7	657.312	1.2266	3586.75±92.88^{c}	3178.32±46.69b	2683±90.63 a	4989.96±46.62d	5174.93±63.81e	5417.88±46.32^{f}
43.	Cyclohexanone	C108941	C6H10O	98.1	1323.9	709.92	1.456	815.31±36.82^{bc}	766.51±46.14^{b}	684.99±20.03^{a}	1141.35±14.29^{e}	942.67±29.73^{d}	847.5±14.62^{c}
44.	Methyl heptenone	C110930	C8H14O	126.2	1346.7	771.224	1.1771	232.83±12.61^{b}	277.96±8.74^{d}	252.4±11.13^{c}	155.84±6.14^{a}	168.00±2.80^{a}	169.43±3.84^{a}
45.	3-Octanone	C106683	C8H16O	128.2	1253.6	549.622	1.3059	241.17±10.79^{a}	194.02±0.79^{a}	239.04±8.25^{a}	1825.19±65.03^{c}	2210.77±7.88^{d}	1417.96±40.12^{b}
46.	2-Nonanone	C821556	C9H18O	142.2	1392.8	912.122	1.4047	206.5±6.33^{b}	190.56±8.31^{b}	155.69±10.06^{a}	400.85±7.53^{e}	336.07±3.23^{c}	371.26±12.41^{d}
47.	Hexan-2-one	C591786	C6H12O	100.2	1126	356.361	1.5097	63.32±1.17^{a}	64.57±11.06^{a}	65.52±5.72^{a}	127.5±16.38^{b}	135.12±0.72^{b}	125.06±1.04^{b}
48.	Ethyl butyrate	C105544	C6H12O	116.2	812.6	154.031	1.1981	845.56±7.69	1034.84±41.	884.8±37.4	488.96±2.01^{a}	584.57±8.44^{b}	675.91±8.60

			2					^{d}	02^{e}	2^{d}			^{c}
49.	1-Methylethyl acetate	C108214	C5H10O2	102.1	843.9	164.532	1.1536	3692.15±36.38^{a}	3680.28±8.01^{a}	3726.91±19.49^{a}	6643.61±60.75^{d}	6156.31±16.06^{c}	6002.76±21.06^{b}
50.	Ethyl 2-methylbutanoate	C7452791	C7H14O2	130.2	1017.4	255.441	1.23	198.67±1.50^{b}	183.1±3.80^{a}	179.75±3.54^{a}	296.24±0.66^{d}	326.58±0.65^{e}	281.35±10.83^{c}
51.	Ethyl acetate	C141786	C4H8O2	88.1	927.6	200.939	1.3279	1170.49±49.74^{a}	1116.83±17.98^{a}	987.75±24.19^{a}	2073.26±142.13^{b}	2706.33±110.28^{c}	2153.36±131.23^{b}
52.	Hexyl acetate	C142927	C8H16O2	144.2	1229.3	505.293	1.4109	83.59±2.97^{a}	77.48±8.60^{a}	76.87±7.47^{a}	848.48±9.51^{b}	847.64±18.34^{c}	1115.77±9.55^{b}
53.	Ethyl octanoate	C106321	C10H20O2	172.3	1415	988.726	1.471	333.27±22.90^{a}	307.3±12.49^{a}	328.16±9.13^{a}	954.27±14.35^{d}	754.11±27.22^{c}	633.23±21.42^{b}
54.	Methyl acrylate	C96333	C4H6O2	86.1	943.7	208.763	1.2691	1548.46±11.06^{c}	1656.27±53.52^{d}	1691.96±27.63^{d}	1089.36±35.06^{a}	1097.63±10.89^{a}	1458.46±8.36^{b}
55.	P-mentha-1,3-diene	C99865	C10H16	136.2	1129.9	360.938	1.21	128.94±1.08^{a}	139.54±3.43^{b}	119.83±5.88^{a}	233.81±5.17^{d}	231.86±2.37^{d}	180.92±8.67^{c}
56.	Alpha-Phellandrene	C99832	C10H16	136.2	1191.3	443.017	1.2233	157.7±3.67^{d}	131.48±2.79^{c}	143.13±9.46^{c}	112.16±1.79^{b}	107.81±10.65^{b}	75.41±4.57^{a}
57.	Styrene	C100425	C8H8	104.2	1230	506.542	1.5069	46.40±4.22^{a}	45.88±2.35^{a}	46.53±2.89^{a}	62.28±2.38^{b}	66.76±1.55^{b}	84.93±0.77^{c}
58.	Limonene	C138863	C10H16	136.2	1196	450.178	1.6555	171.72±2.78^{a}	171.62±8.32^{a}	167.74±4.98^{a}	247.16±10.62^{b}	334.76±7.41^{c}	645.73±17.28^{d}
59.	Methyl pyrazine(M)	C109080	C5H6N2	94.1	1280	605.331	1.0806	4104.02±114.05^{d}	3710.12±98.32^{c}	3703.55±50.22^{c}	2767.77±44.36^{a}	2641.57±17.18^{a}	3207.16±109.21^{b}
60.	Methyl pyrazine(D)	C109080	C5H6N2	94.1	1277.9	600.848	1.3886	2683.83±119.54^{b}	2105.55±132.42^{a}	2001.31±15.89^{a}	8146.84±185.41^{c}	9555.21±132.30^{d}	9447.13±30.89^{d}
61.	2,5-	C123320	C6H8N2	108.1	1312.	681.908	1.1134	2558.67±22.	2228.3±21.4	2439.99±44	855.69±10.3	746.85±9.46^{b}	642.21±30.2

	Dimethylpyrazine (M)				8			11^{f}	6^{d}	.40^{e}	6^{c}		2^{a}
62.	2,5-Dimethylpyrazine (D)	C123320	C6H8N2	108.1	1311.5	678.661	1.4976	900.05±22.12^{d}	730.28±34.08^{ab}	763.14±15.48^{b}	837.51±4.42^{c}	893.82±14.72^{d}	717.67±5.16^{a}
63.	Trimethylpyrazine	C14667551	C7H10N2	122.2	1353.1	789.49	1.1694	331.24±9.97^{c}	376.61±9.43^{d}	331.36±20.51^{c}	219.19±8.05^{b}	192.84±2.04^{a}	176.67±5.19^{a}
64.	2,5-Dimethylfuran	C625865	C6H8O	96.1	967.9	222.541	1.0216	500.37±10.71^{a}	484.23±25.19^{a}	490±14.11^{a}	1181.64±1.11^{d}	997.58±0.35^{c}	937.76±35.61^{b}
65.	2-Ethylfuran	C3208160	C6H8O	96.1	961.4	218.728	1.2978	304.5±7.63^{a}	303.77±6.51^{a}	332.15±18.57^{b}	432.85±6.81^{d}	391.07±2.70^{c}	459.59±3.36^{e}
66.	2-Methyl-3-(methylthio)furan	C63012975	C6H8OS	128.2	1332.4	732.249	1.1088	1808.44±29.67^{cd}	1852.94±38.68^{d}	1717.69±94.71^{c}	1367.05±63.61^{b}	1162.43±25.10^{a}	1220.27±57.04^{a}
67.	Dimethyldisulfide	C624920	C2H6S2	94.2	1029.1	264.273	1.1459	206.70±3.29^{d}	151.91±1.34^{a}	202.35±7.12^{d}	166.94±10.14^{b}	149.34±0.42^{a}	190.61±2.96^{c}
68.	Dimethyl trisulfide	C3658808	C2H6S3	126.3	1415.7	991.254	1.2947	238.85±5.34^{a}	234.06±11.49^{a}	267.68±7.40^{b}	454.03±14.39^{c}	495.02±19.36^{d}	484.02±3.34^{d}
69.	Ethane, 1,1-diethoxy-	C105577	C6H14O2	118.2	897.6	186.049	1.0335	2622.71±33.65^{d}	2723.2±16.02^{e}	2644.27±49.39^{d}	2161.58±11.88^{b}	2487.68±50.38^{c}	1835.59±18.51^{a}
70.	2,2,4,6,6-Pentamethylheptane	C13475826	C12H26	170.3	917.9	195.501	1.3308	3111.08±31.68^{c}	2919.48±4.02^{a}	3040.61±28.94^{b}	4069.16±44.46^{e}	3431.78±20.85^{d}	3471.43±29.68^{d}
71.	Toluene	C108883	C7H8	92.1	1056.2	286.427	1.0276	832.56±13.77^{ab}	821.98±7.10^{ab}	785.28±22.29^{a}	939.56±85.93^{c}	818.19±6.99^{ab}	887.92±21.28^{bc}
72.	Cis-rose-oxide	C303323	C10H18	154.3	1338.	752.709	1.368	236.25±28.2	197.22±16.2	182.12±6.2	1364.35±78.	1445.82±8.4	1291.26±11

		6	O		4			2^{a}	2^{a}	8^{a}	77^{bc}	3^{c}	2.96^{b}
73.	2-Butoxyethanol	C111762	C6H14O2	118.2	1444	1098.489	1.2096	375.09±6.33^{a}	350.12±9.12^{a}	300.4±5.20^{a}	1796.97±124.69^{b}	2247.34±75.32^{c}	1950.99±166.19^{b}
74.	Triethylamine	C121448	C6H15N	101.2	803.7	151.294	1.211	839.86±5.68^{f}	665.28±22.44^{d}	792.29±15.82^{e}	581.01±3.79^{b}	536.04±6.07^{a}	608.2±8.18^{c}
75.	Acetic acid	C64197	C2H4O2	60.1	1466.2	1190.827	1.1489	1243.71±76.19^{a}	1147.42±17.20^{a}	1142.56±35.43^{a}	4937.91±604.78^{b}	6903.21±238.67^{c}	5301.99±541.02^{b}
76.	2,5-Dimethylthiophene	C638028	C6H8S	112.2	1142.4	376.023	1.075	223.7±11.80^{a}	197.63±4.67^{a}	209.19±6.04^{a}	705.3±22.55^{b}	601.71±16.11^{c}	578.07±16.81^{b}
